# The cytogenetic aspect of male infertility

**Published:** 2014-12

**Authors:** Fatima Ammar-Khodja, Zohra Hamouli, Fella Boukerbout F, Karima Djerroudib

**Affiliations:** 1 *Unit of Genetics, Laboratory of Molecular and Cellular Biology, Faculty of Biological Sciences, University of Sciences and Technology Houari Boumediene, Agiers, Algeria.*; 2 *Unit of Endocrinoly, Laboratory of Population and Organists Biology, Faculty of Biological Sciences, University of Sciences and Technology Houari Boumediene, Agiers, Algeria.*

**Dear Editor**

Infertility affects approximately 15% of couples worldwide. Within 50% of cases, man provides reproductive function disorders (1). The cause of infertility in men with oligospermia and azoospermia seems to be due to underlying genetic abnormalities (2). Chromosomal abnormalities are one of the causes of human infertility as they interfere with spermatogenesis. The frequency of chromosomal aberrations and specific translocations in infertile men is multiplied by 10 compared with the normal population (3). Hundred patients aged between 26 and 50 years (the middle age 35 years old), oriented by specialized medical structures, are included in our study. All patients were referred for sterility (no spontaneous pregnancy despite >1 year unprotected intercourse). Only couples with infertility primary, in which men had a review of abnormal sperm: azoospermia or severe oligospermia (concentration sperm cells <5×10^6^ ml and mobility <40%) were included in the study. 20 metaphases for each patient were analyzed by GTG banding technique (Giemsa Trypsin G bands).

**Figure 1:**
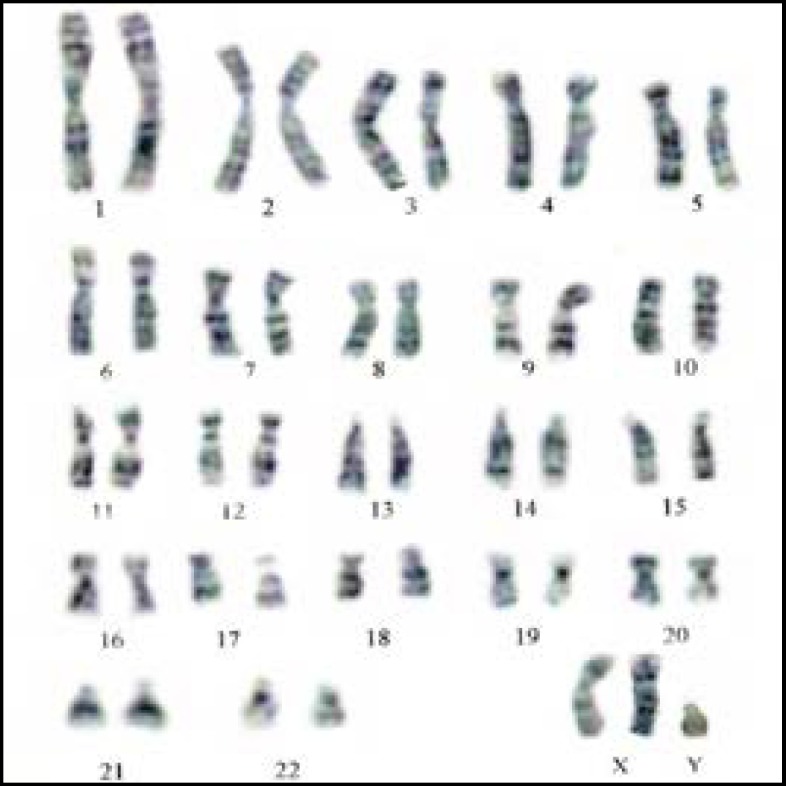
Karyotype of a syndrome Klinefelter: 47,XXY

**Figure 2:**
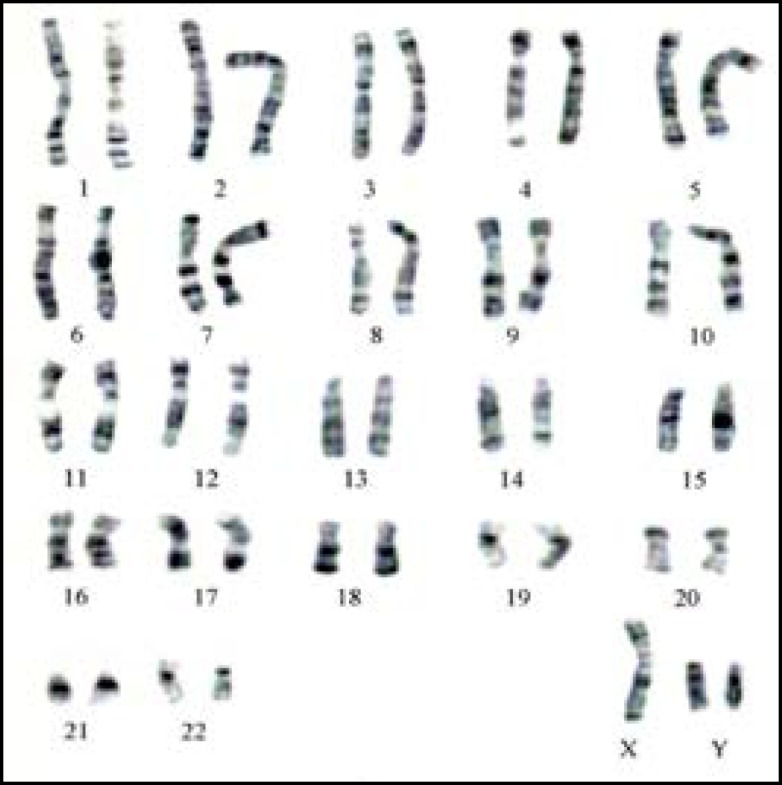
Karyotype of aneuploidy 47,XYY

**Figure 3:**
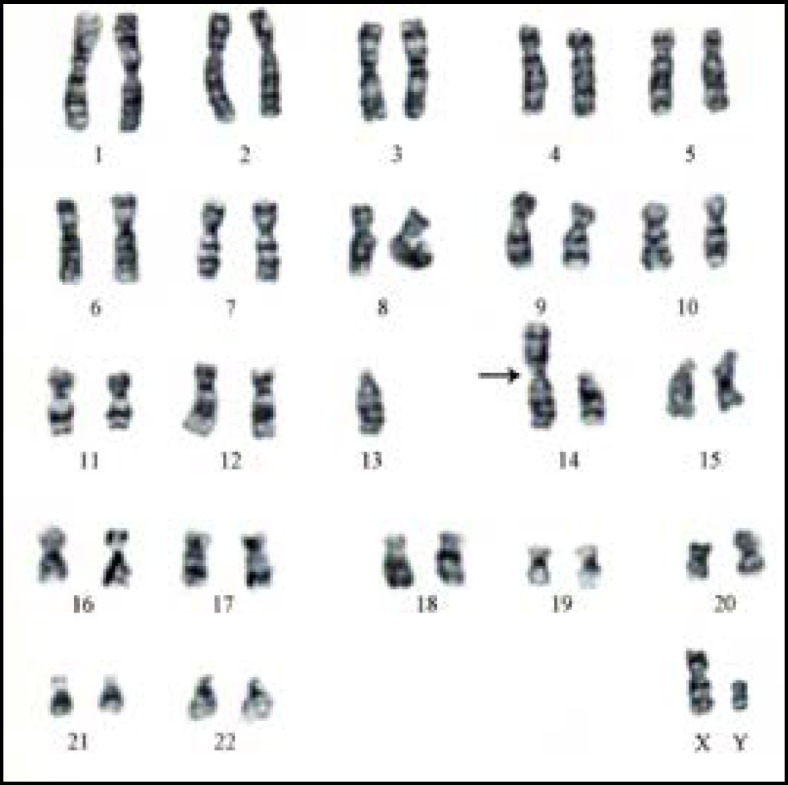
Karyotype 45,XYder(13)(14)

**Figure 4:**
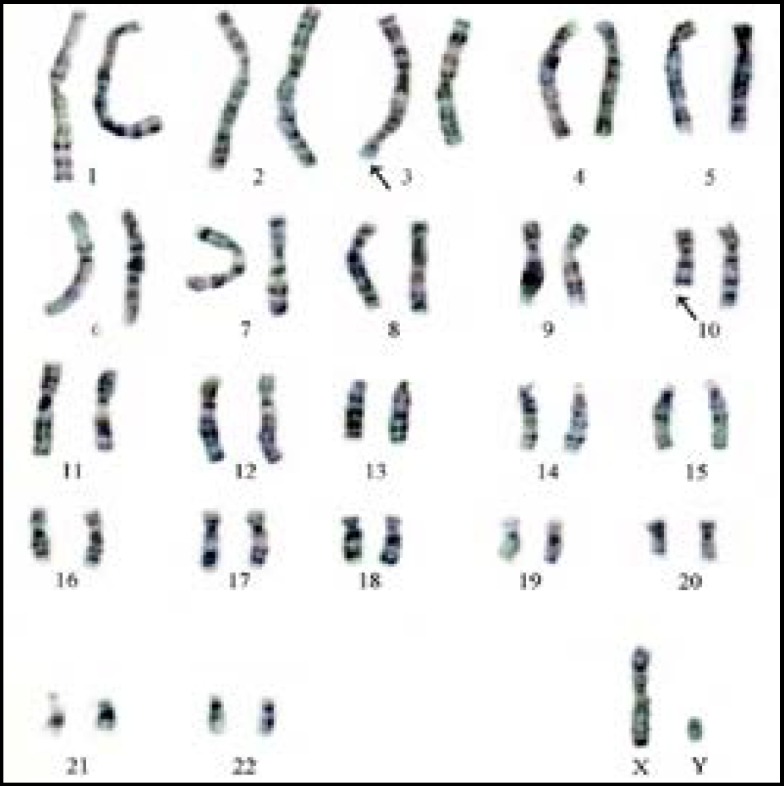
Karyotype 46, XY t(3q)(10q)

In our sample, there were 76 patients (76%) with an ordinary karyotype of which 5 have a known etiology. The most frequent medical history was a mumps orchitis, testicular ectopia, right and left inguinal testicles, or a bilateral varicocele. For patients without obvious etiology, it was important to mention the environmental and other genetic factors unidentified within the limits of the used technique. The chromosome abnormality rate was 24%; the numerical type 21% and structural type 3%. The chromosomal aberrations found in this study, were gonosomal (21/24: 87.5%) and autosomal 3/24: 12.5%). In 64.2% (18/28) of patients the azoospermia was determined by aneuploidy 47, XXY (Figure 1). Subjects 47, XXY (18/100 patients: 18%) had clinical signs or a complete picture mentioning Klinefelter syndrome. Aneuploidy 47, XYY was identified in three patients with the oligoasthénospermia (Figure 2). The Robertsonian translocations 45,XY,der (13)(14) (Figure 3) and reciprocal translocation 46, XY t(3q-10q) (Figure 4) explain oligozoospermia and oligoasthenoteratospermia. These findings are in accordance with those from other surveys and confirm that the XXY aneuploidy is the most frequent chromosomal abnormality in azoospermic individuals. The correlation is established between the karyotypic abnormalities and sperm characteristics.

A review of the literature of somatic chromosome investigations in infertile males has shown that 10-15% of azoospermic males and 4-5% of oligozoospermic males have an abnormal karyotype. In the first group, sex chromosome abnormalities predominate (mainly 47,XXY), whereas in the latter, autosome anomalies (i.e. Robertsonian and reciprocal translocations) are the most frequent. However, the prevalence of somatic chromosomal abnormalities in male sterile population has been reported and varies in different studies and populations. For example, the prevalence of chromosomal abnormalities in Dutch or Italian infertile population is respectively 3.1% and 3.95 (4). In Iran, cases with an abnormal karyotype were 13.96% (5). The frequency of chromosome abnormalities appears higher in some countries. Thus, this frequency in infertile men from western Mexico and eastern Turkey is respectively 18.9% and 23.26% (6, 7). In Estonia, total chromosome alterations were revealed in 47.8% of infertile men (8).

Interestingly, our present study shows a higher percentage of chromosomal abnormalities (24%) in comparison with previous reports where the range was between 10 and 15% (9). These frequency variations can be explained by the choice of recruitment criteria based on the results of spermiograms (azoospermia or severe oligospermia: concentration sperm cells <5×10^6^ ml). The abnormal gonosome number (XXY) in patients with azoospermia was higher than in patients with severe oligozoospermia. Our results confirm the data of different literature sources that certify that sex chromosome abnormalities are the privilege of azoospermia. Indeed Klinefelter's syndrome in its homogeneous form (47, XXY) representing the most common aneuploidy (85.7%: 18/21). 47,XYY is a sex chromosomal abnormality observed in humans, with a prevalence of 0.1% of male births. This abnormality can be characterized by oligoasthenoszoospermia and it’s relatively uncommon and can miss clinically because of its variable clinical presentations (10). Our study reported a higher percentage of 3%. The reciprocal translocations are the most frequent structural chromosomal anomalies in oligozoospermia). In our study, two patients with Robertsonian translocation 45, XY, der (13)(14) were suffering from oligospermia. The balanced translocation 46, XY t(3q)(10q) has been identified in one patient with oligoasthenozoospermia.
